# Functional characterization of SLC39 family members ZIP5 and ZIP10 in overexpressing HEK293 cells reveals selective copper transport activity

**DOI:** 10.1007/s10534-022-00474-6

**Published:** 2022-12-01

**Authors:** Marcello Polesel, Alvaro Ingles-Prieto, Eirini Christodoulaki, Evandro Ferrada, Cédric Doucerain, Patrick Altermatt, Michelle Knecht, Michael Kuhn, Anna-Lena Steck, Maria Wilhelm, Vania Manolova

**Affiliations:** 1 Vifor (International) AG, Wagistrasse 27a, 8952 Schlieren, Switzerland; 2 CeMM Research Center for Molecular Medicine of the Austrian Academy of Sciences, Lazarettgasse 14, 1090 Vienna, Austria

**Keywords:** SLC39A5, SLC39A10, ZIP5, ZIP10, Ionomics, ICP-MS

## Abstract

Zinc is the second most prevalent metal element present in living organisms, and control of its concentration is pivotal to physiology. The amount of zinc available to the cell cytoplasm is regulated by the activity of members of the SLC39 family, the ZIP proteins. Selectivity of ZIP transporters has been the focus of earlier studies which provided a biochemical and structural basis for the selectivity for zinc over other metals such as copper, iron, and manganese. However, several previous studies have shown how certain ZIP proteins exhibit higher selectivity for metal elements other than zinc. Sequence similarities suggest an evolutionary basis for the elemental selectivity within the ZIP family. Here, by engineering HEK293 cells to overexpress ZIP proteins, we have studied the selectivity of two phylogenetic clades of ZIP proteins, that is ZIP8/ZIP14 (previously known to be iron and manganese transporters) and ZIP5/ZIP10. By incubating ZIP over-expressing cells in presence of several divalent metals, we found that ZIP5 and ZIP10 are high affinity copper transporters with greater selectivity over other elements, revealing a novel substrate signature for the ZIP5/ZIP10 clade.

**Supplementary Information:**

The online version contains supplementary material available at 10.1007/s10534-022-00474-6.

## Introduction

Zinc is the second most prevalent metal element present in eukaryotic living organisms, and control of its concentration is pivotal to cellular biology (Kambe et al. 2021). Zinc in living systems is mostly found in protein complexes, thanks to its unique coordination chemistry (Rosenzweig 2002; Andreini et al. 2006) and serves as a cofactor in a vast number of cellular processes including replication (MacDonald 2000), signaling (Hojyo and Fukada 2016), vesicle trafficking and secretory activities (Kambe et al. 2017; Chen et al. 2000; Lefebvre et al. 2012). The amounts of zinc available to the cell cytoplasm and within the various cellular organelles are tightly controlled and the activity of zinc transporters belonging to the SLC30 and SLC39 families, also known as Zinc transporters (ZnTs) and Zrt/Irt-like proteins (ZIPs), respectively, ensure the regulation of cellular zinc concentrations (Hojyo and Fukada 2016; Palmiter and Huang 2004; Eide 2004; Eide 2004). These two families of solute carrier proteins (SLCs) play a crucial role in homeostasis, and emerging evidence from human genetics studies links polymorphisms in these two gene families to various human diseases (Flannick et al. 2014; Wang et al. 2002; Chowanadisai et al. 2006; Giunta et al. 2008; Anzilotti et al. 2019), and points to the evaluation of these SLC proteins as pharmacological targets (Hara et al. 2022). Selectivity of ZIPs membrane transporters has been the focus of numerous studies on the mammalian zinc transporters measuring metal uptake into proteoliposomes and provided preliminary evidence for substrate selectivity of ZIPs to zinc over other metals such as copper, iron, and manganese (Lin et al. 2010). The structural features responsible for zinc permeability have been fully elucidated from observations carried out on the bacterial homologue of ZIP proteins, ZIPB, of which crystal structure is available (Zhang et al. 2017a). The mammalian SLC39/ZIP family has an ancient evolutionary history, and its members are broadly conserved amongst species, with some clustering together as evidenced by sequence alignments, like members of the LIV-1-type ZIP transporters (Hu 2021). Sequence similarities have shown shared structural and functional features, including substrate specificity. For example, SLC39A8 (ZIP8) and SLC39A14 (ZIP14), display phylogenetic vicinity and are iron and manganese transporters, as numerous functional studies have provided a view on their physiological role in iron and manganese homeostasis (Sterling et al. 2017; Wang et al. 2012; Lin et al. 2017; Scheiber et al. 2019). In contrast, SLC39A5 (ZIP5) and SLC39A10 (ZIP10) have long been postulated to be zinc-specific, and it is not known if they may transport any additional metal substrates. Here, we have studied the transport capacity of these two phylogenetically LIV-1 clusters, that is ZIP8/ZIP14 (previously known to be iron and manganese transporters) and ZIP5/ZIP10. By incubating HEK293 over-expressing cells with concentrated extracellular divalent metals, we found that ZIP5 and ZIP10 are high affinity copper transporters with no selectivity over other elements, suggesting a novel signature transport property for the two members of the SLC39 family.

## Results

### Quantification of divalent metal transport through ZIP5, ZIP8, ZIP10 and ZIP14

**Fig. 1:**
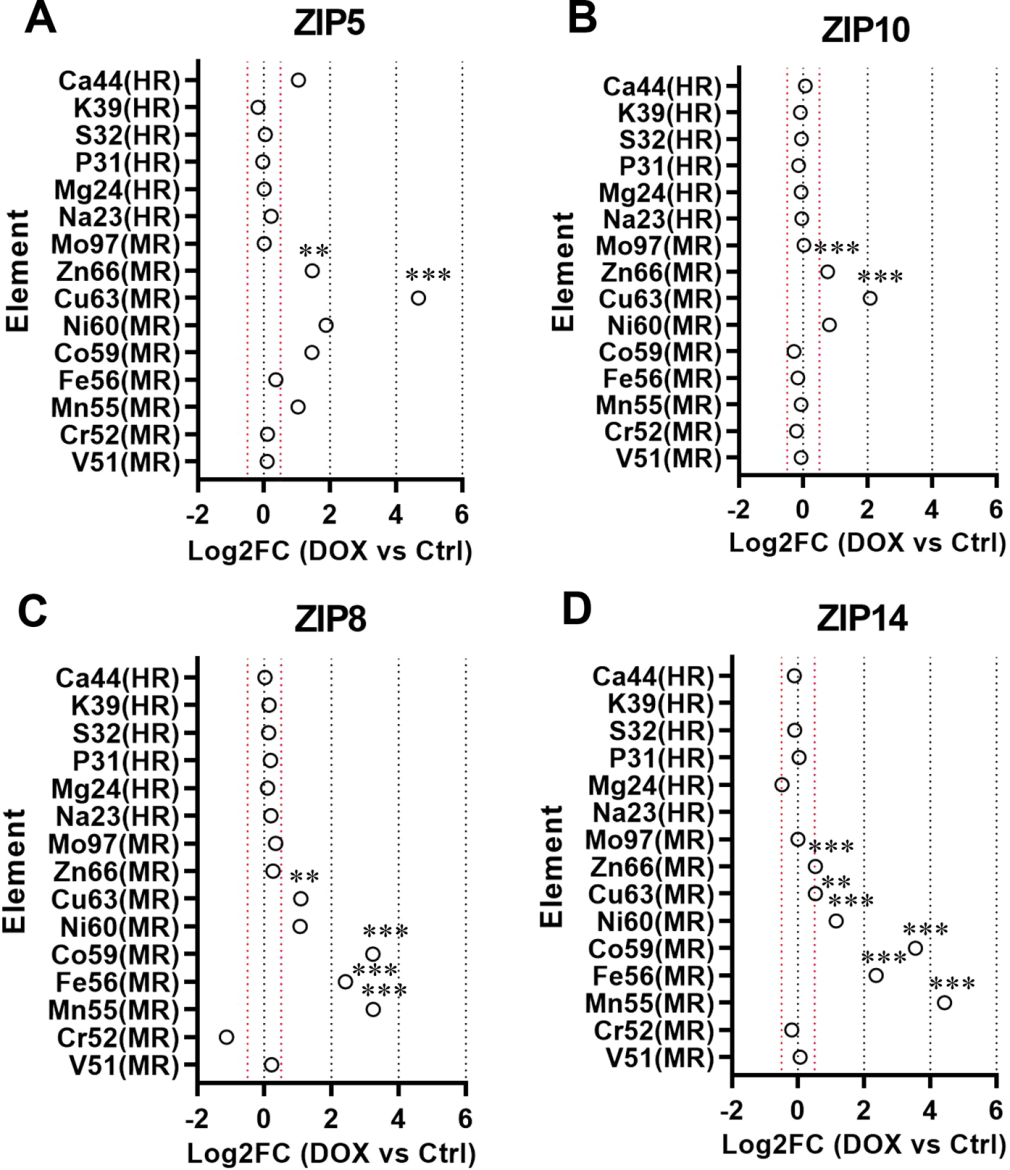
The plots show the quantification of the concentrations measured in overexpressing cells (DOX) for the indicated elements analyzed by ICP-MS and expressed as log2 fold change against control (Ctrl) cells. Ion concentrations for each element were normalized to the total protein concentration in the respective cell lysate. ICP-MS analysis of cell lysates showed a significant increase of Cu63 from HEK cells overexpressing ZIP5 (**A**) and ZIP10 (**B**). Mn55, Co59 and Fe56 were found to have increased in ZIP8 (**C**) and ZIP14 (**D**). The red dashed line represents the significance threshold, corresponding to a value of log2 fold change of 0.5 (at least 70% difference in ± DOX) (N = 3 biological replicates per group ± DOX.**p < 0.01;***p < 0.001). (Color figure online)

We made use of inducible HEK293-JumpIn-ZIP5,8,10,14-overexpressing cells that were produced and characterized as described previously (Sijben et al. 2021). We first confirmed localization of the transgene product to the plasma membrane by immunofluorescence (Online Resource 1). Localization of the overexpressed ZIP protein to the plasma membrane was necessary to detect uptake of metals in cell lysates. To screen for uptake properties amongst ZIPs, we incubated HEK293 ZIP5, ZIP8, ZIP10 and ZIP14 overexpressing cells with a cocktail of divalent metals including iron, zinc, copper, manganese, magnesium, cobalt and nickel (Fig. 1). As a control, we tested all ZIPs over-expressing cells incubated with buffer only (Online Resource 2). To exclude relevant biases arising from dysregulation of other SLC39 family transporters, we performed RNA sequencing of our HEK293 ZIP5, ZIP8, ZIP10 and ZIP14 overexpressing cells and results are available online (Online document. RESOLUTE Transcriptomics Dashboard 2022). In this setting, ZIP8 and ZIP14 showed increases in ^55^Mn and ^56^Fe as well as increases in non-physiological metals ^59^Co and ^60^Ni in lysates of over-expressing versus control cells (Fig. 1C, D). In contrast, we found elevated levels of ^63^Cu in lysates of ZIP5 and ZIP10 overexpressing cells (Fig. 1A, B). ^66^Zn was found to be elevated in all four groups, but the difference between induced and uninduced cells was not as prominent.

### Copper competes with zinc for cellular influx through ZIP5 and ZIP10

**Fig. 2:**
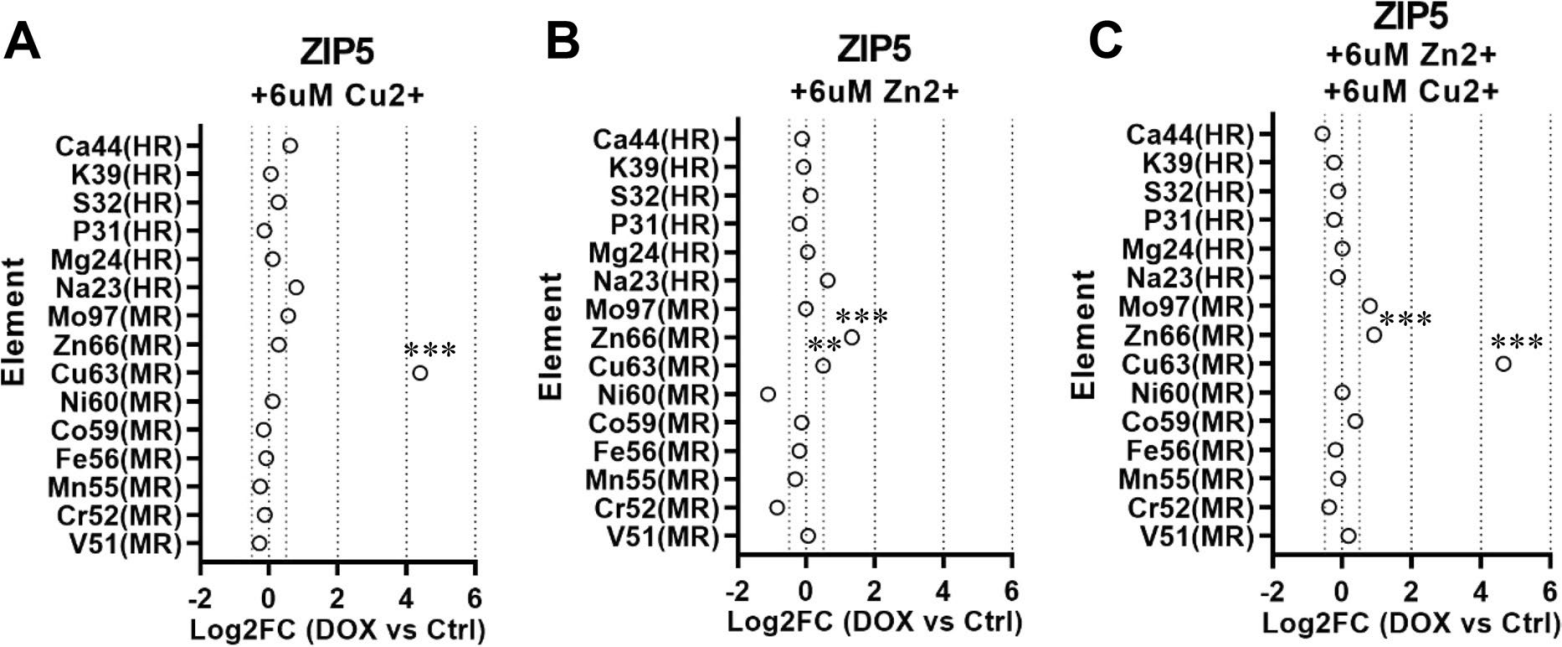
**A**–**C** The plots show the quantification of the concentrations measured in overexpressing cells (DOX) for the indicated elements analyzed by ICP-MS and expressed as log2 fold change against control (Ctrl) cells. Ion concentrations for each element were normalized to the total protein concentration in the respective cell lysate. ICP-MS analysis of cell lysates showed a significant increase of Cu63 from HEK cells overexpressing ZIP5. The increase (log2FC in DOX + vs DOX- > 4) is equal when cells are incubated with Cu^2+^ and with equimolar amounts of Cu^2+^ and Zn^2+^, suggesting higher affinity for Cu^2+^. The red dashed line represents the significance threshold, corresponding to a value of log2 fold change of 0.5 (at least 70% difference in ± DOX) (N = 3 biological replicates per group ± DOX. **p < 0.01; ***p < 0.001). (Color figure online)

**Fig. 3:**
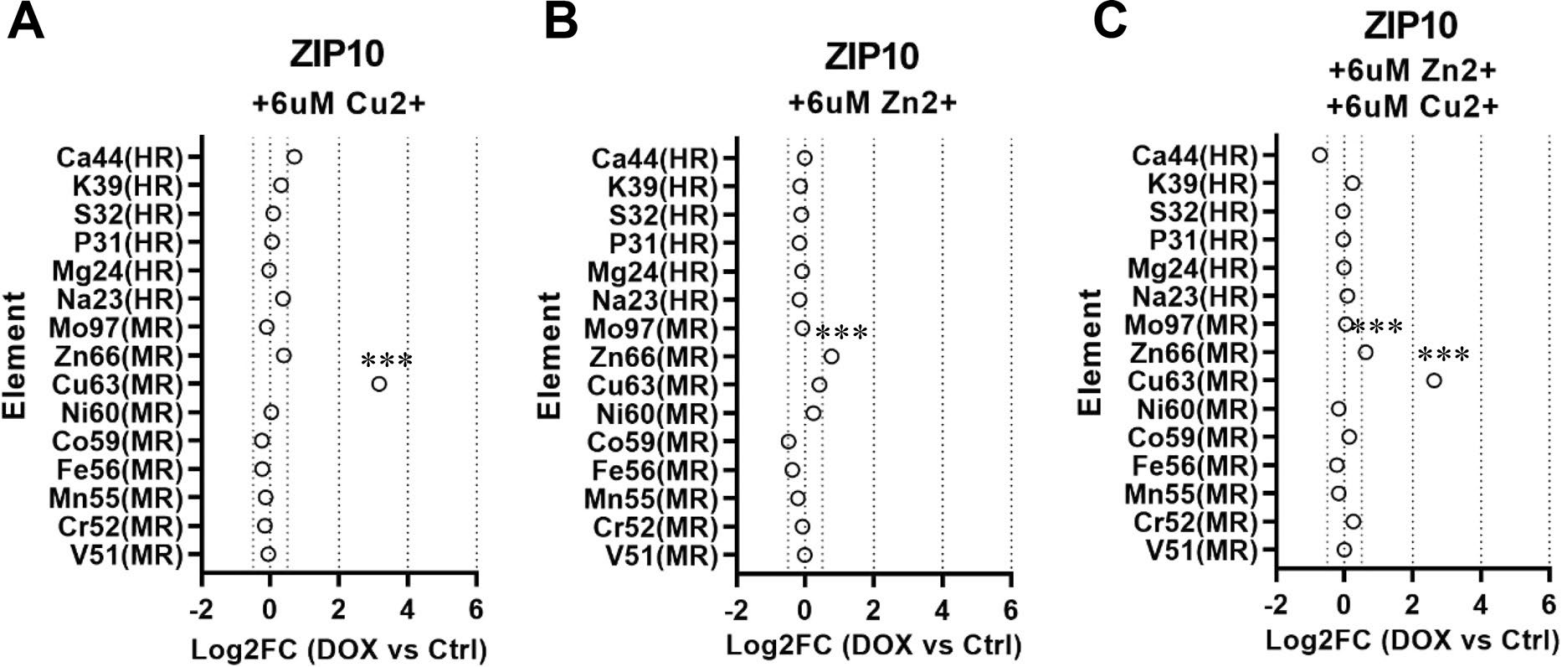
**A**–**C** The plots show the quantification of the concentrations measured in overexpressing cells (DOX) for the indicated elements analyzed by ICP-MS and expressed as log2 fold change against control (Ctrl) cells. Ion concentrations for each element were normalized to the total protein concentration in the respective cell lysate. ICP-MS analysis of cell lysates showed a significant increase of Cu63 from HEK cells overexpressing ZIP10. The increase (log2FC in DOX + vs DOX- > 2) is equal when cells are incubated with Cu^2+^ and with equimolar amounts of Cu^2+^ and Zn^2+^, suggesting higher affinity for Cu^2+^. The red dashed line represents the significance threshold, corresponding to a value of log2 fold change of 0.5 (at least 70% difference in ± DOX) (N = 3 biological replicates per group ± DOX. **p < 0.01; ***p < 0.001). (Color figure online)

Next, we sought to investigate whether copper and zinc transport through ZIP5 and ZIP10 happen competitively, and to test affinity of these two zinc transporters for copper. We therefore incubated cells with equal amounts of the two divalent metals, and with either of the two to see how this could impact the measured influx. We found that incubation with zinc only led to a minimal increase in ^66^Zn as measured by ICP-MS in ZIP5 and ZIP10 over-expressing cell lysates (Figs. 2B, 3B). In contrast, incubation with copper only, or equimolar amounts of copper in combination with zinc led to abrupt increases in ^63^Cu influx and decreased ^66^Zn amounts (Figs. 2A, C, 3A, C). When incubated with high amounts of copper, ZIP8 and ZIP14 over-expressing cell lysates showed moderate increase in quantified ^63^Cu, similar to the increase observed for their substrates ^55^Mn and ^56^Fe that were not spiked in (Online Resource 3).

**Fig. 4:**
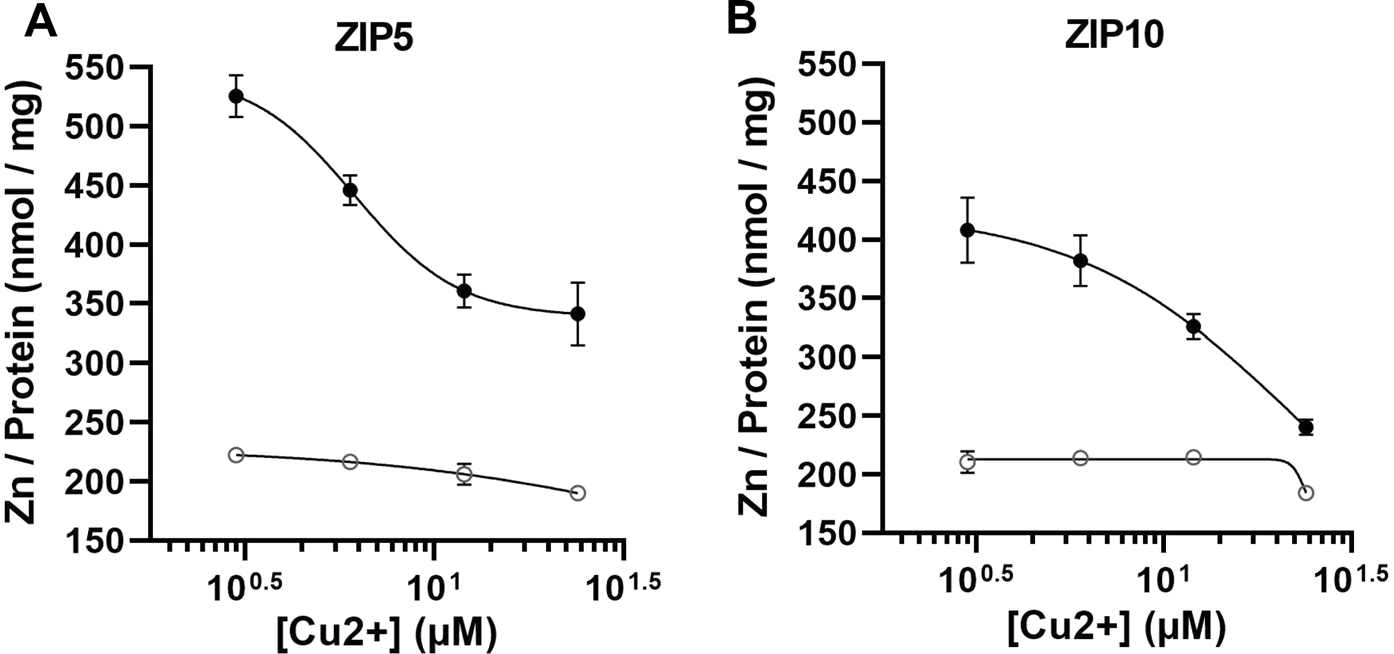
Displacement curves for copper against zinc in HEK293 ZIP5 (**A**) and ZIP10 (**B**) over-expressing cells. In these experiments, increasing concentrations of copper were administered (0, 3, 6, 12 and 24 μM) to cells and co-incubated with 6 μM zinc. To facilitate the reader, zinc concentration at 0 μM Cu^2+^ is not displayed. Copper dose-dependently inhibited uptake of zinc through ZIP5 (IC50:1.93 μM) and ZIP10 (IC50:76.40 μM) in DOX-treated cells (dark dots) and not in uninduced, vehicle-treated cells (white dots). (Color figure online)

Collectively, these results show that ZIP5 and ZIP10 have greater selectivity for copper than for zinc, and that elevated copper levels can shut down zinc transport. In contrast, ZIP8 and ZIP14 over-expressing cells show no or little increased influx of copper and zinc, respectively, even when the two substrates are provided alone and in high concentrations. Zinc uptake inhibition experiments were carried out, in which copper was allowed to compete with a fixed concentration of zinc (6 μM) (Fig. 4). In HEK293 ZIP5 and ZIP10 overexpressing cells, copper most easily displaced zinc from transport as evidenced by the reduction of zinc content in lysates when cells were incubated with increasing amounts of copper (Fig. 4A, B).

## Discussion

The physiological relevance of zinc as the second most abundant trace element in the human body is evidenced by the fact that almost 10% of the human genome codes for zinc-binding proteins (Andreini et al. 2006). Zinc’s redox properties make it particularly adapted to serve as a co-factor of enzymes and as a structural component determining tertiary folding of proteins (Carter et al. 2014). On the other hand, copper is the third most abundant trace element in the human body and serves as an essential element for enzymes involved in various biochemical processes such as in aerobic metabolism in the mitochondria, and in the protection against free-radical damage (Kim et al. 2008). Various genetic conditions related to defects in genes involved in dietary absorption (Rodriguez-Castro et al. 2015; Tumer and Moller 2010) lead to dysregulation of zinc and copper levels in the body and consequently to organ insufficiency which translates into various rare conditions characterized by mental retardation, anemia, neutropenia, and cardiac hypertrophy. Conversely, elevated zinc or copper levels are toxic, and mammals have adapted an integrated network of zinc-, and copper-binding proteins and transporters to take up, efflux, compartmentalize it and maintain their levels within controlled ranges. Zinc and copper homeostasis have been so far seen as two discrete processes, and the proteins responsible for their body storage, transport and excretion have been independently defined as two separate entities. Indeed, zinc transporters belonging to the SLC30 and SLC39 families have been associated with the uptake and regulation of cellular zinc concentrations. On the other hand, body copper levels have so far been defined to be controlled by members of the SLC31 family and P-Type ATPases (Veldhuis et al. 2009). At present, it is not reported to what extent the two transport systems overlap and whether such a case is of physiological relevance. Therefore, functional studies on this topic will help in better understanding zinc and copper homeostasis.

In our study, we focused on investigating the metal selectivity of LIV-1 members ZIP5, ZIP8, ZIP10 and ZIP14 proteins. To do so, we engineered HEK293 cells to overexpress the human version of these four ZIP proteins and quantitatively analyzed the composition of cell lysates previously incubated with divalent metals as zinc, copper, iron and manganese with inductively coupled plasma-mass spectrometry (ICP-MS).

The advantage of using the human embryonic kidney 293 Jump In T-Rex cell system (HEK293-JI) is that it allows for effortless introduction of genetic material and inducible expression of the retargeted gene, while providing isogenic expression of the target protein from a defined genomic locus. Intracellular proteins were particularly difficult to characterize functionally in our assay set-up, as lysates of cells incubated in a metal-containing media in such cases did not show the expected uptake patterns (unpublished data). Although this limitation could be technically overcome by redirecting ZIP proteins to the plasma membrane (Dvorak et al. 2021), redirection could also lead to modification of cellular metal gradients, and result in alterations. Instead, investigation of transport of metals provided extracellularly through ZIP proteins required adequate plasma-membrane expression. Therefore, we inserted an HA-Tag at the C-terminus portion of the ZIP sequence, in order to verify transgene overexpression efficiency and distribution across cells, its expression levels and its plasma-membrane cellular localization (see “Materials and Methods”).

To assess the function of ZIP5, ZIP8, ZIP10 and ZIP14 in our HEK293-JI cellular system, ionomics screening was performed. This technique is the golden standard in plant elemental analysis due to its sensitive, cost-effective, and reproducible characteristics (Baxter 2010). Ionomics has been previously used to measure trace element composition of animals (Ma et al. 2015), and we recently described the use of this technique to investigate the intracellular ionome of HEK293 cells (Dvorak et al. 2021). In this study, we measured the metal content in ZIP over-expressing cells by providing them with predetermined concentrations of metal-chloride salts and determining uptake over a time course of 3 h upon induction of the transgene. We found that ZIP5 and ZIP10 are copper transporters as evidenced by conspicuous increases in ^63^Cu levels in lysates of cells incubated with a metal mix containing zinc, copper, iron and manganese (Fig. 1). Competition experiments using fixed zinc concentrations and increasing amounts of copper validated that these proteins can permeate both elements (Fig. 4).

Our experiments validate ionomics as a method to study trace element selectivity in mammalian cells engineered to overexpress plasma-membrane localized metal transporters, however there are limitations that deserve careful consideration. First, although our method is label-free and does not require radiolabeled substrates, the solute carrier-type mediated transport of metal ions across the plasma membrane remains subjected to electrochemical gradients that are maintained in live cells and might interfere with the readout. Second, metal elements are already present at different concentration ranges in living cells. As seen in the control cells that are not treated with doxycycline to induce transgene overexpression, baseline concentration of ^66^Zn is higher than this of ^63^Cu, and this makes a direct evaluation of what is the preferred substrate impossible in our set-up. Third, ICP-MS is not able to detect the copper oxidation state that is transported through these SLCs. In biological systems, copper is cycling between two major oxidation states Cu(I) and the more labile Cu(II). Several approaches have been developed to detect Cu(I) in living cells (Ackerman et al. 2017), and recently a new chemical sensor that detects Cu(II) was developed to identify the copper oxidation state transported by CTR1 and DMT1 (Pezacki et al. 2022). These sensors could be employed in future studies to identify the copper oxidations states transported by the ZIP proteins.

**Fig. 5:**
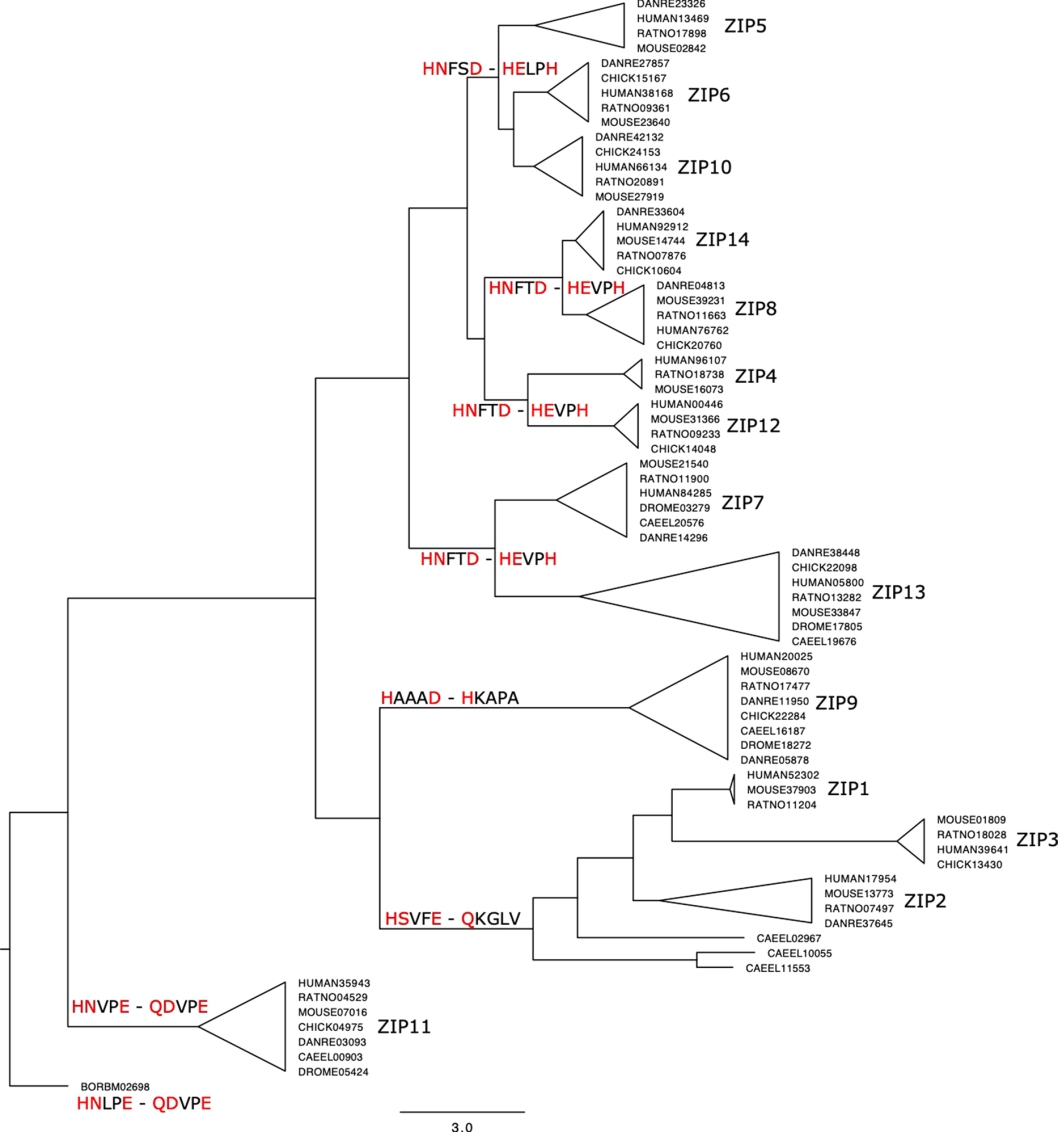
Phylogenetic tree of ZIP family members. The tree was constructed using ZIP orthologs from seven representative species of metazoan (i.e., human, mouse, rat, zebrafish, *c. elegans* and the fruit fly), plus the BbZIP gene from Bordetella sp. (see Methods). Clades were collapsed to ease visualization and labeled at the tips according to the respective ZIP family member. Metal binding motifs M1 (TM4) and M2 (TM5) specific to members of closely related clades are label at the nodes as: “M1–M2”. Residues responsible for metal coordination are highlighted in red. (Color figure online)

Two main binding sites have been previously identified in the crystal structure of BbZIP, a bacterial homolog of the ZIP family (Zhang et al. 2017b), which transports zinc and cadmium. Overall, the two metal binding motifs (M1 and M2) responsible for substrate specificity share the following signatures HNxxE/D and HExPH, and are in spatial proximity in the transmembrane domains (TM) 4 and 5, respectively. These motifs are relatively conserved through members of the ZIP family (Hu 2021). It has been shown, for instance, that most mutations at the equivalent sites in the human SLC39A4 (hZIP4) are loss-of-function mutations (Zhang et al. 2017b, 2020) To investigate whether amino acid substitutions in these motifs might explain the changes in specificity observed in our results, we performed an evolutionary analysis using sequences spanning all ZIP members present in 8 representative species (Fig. 5; see Methods). The resulting phylogenetic tree reveals six major clades with closely related metal binding motifs. ZIP1, ZIP2 and ZIP3 form a closely related clade with binding motifs that depart considerably from the overall ZIP family signature (i.e. HSVFE and HKGLV). Similar to ZIP7/ZIP13; the ZIP8/ZIP14 clade belongs to a separate orthologous group. Although ZIP7 and ZIP13 have identical M2s, the M1 of ZIP13 has swapped the His and Asp residues, at positions 232 and 228. In contrast, ZIP8 and ZIP14 share identical metal binding motifs, and interestingly, the first His residue in the M2 motif has been substituted by a Glu, revealing a common signature, most likely responsible for the differential specificity of ZIP8 and ZIP14 for iron and manganese. Similarly, ZIP5, ZIP6 and ZIP10, form a closely related clade. All three members share a common M2 motif (i.e. HELPH), while M1, although preserved between ZIP10 and ZIP6 (i.e. HNFSD), shows two substitutions in ZIP5 (i.e. HNLTD). These motifs, which are specific to the ZIP5/ZIP10 clade, are most likely responsible for the copper specificity of these transporters, and further suggest that the ZIP6 transporter should also have a preference for copper. However, we could not test ZIP6 in ionomics, given that tagged ZIP6 was not localized on the plasma membrane in HEK293 overexpressing cells.

In conclusion, SLC39 family members (ZIP proteins) play a role in regulating the concentration of several metals present in eukaryotic cells. We investigated the ionome of HEK293 cells engineered to overexpress ZIP8 and ZIP14 proteins, which have previously been shown to selectively transport manganese and iron. We contrasted our finding to ZIP5 and ZIP10 overexpressing cells, previously known to only transport zinc, and found that ZIP5 and ZIP10 are high affinity copper transporters with greater selectivity over other elements, revealing a novel substrate signature for these two transporters. Consistently, ZIP5 and ZIP10 were previously shown to contain PrP-like domains in its structures, and these domains bind copper with high affinity (Pocanschi et al. 2013; Watts et al. 2009). A phylogenetic analysis of all ZIP family members revealed specific signatures in two previously characterized metal binding motifs, revealing amino acid substitutions in these motifs that are specific to ZIP5 and ZIP10, and most likely are responsible for copper specificity. Moreover, the analysis suggests that ZIP6 is most likely a copper transporter. Our findings add value to the ongoing deorphanization effort in the field of metal transporters biology and shine light on substrates other than zinc. Further studies should aim at addressing the biological meaning of ZIP5- and ZIP10-mediated copper transport in vivo, and their involvement in copper pathophysiology.

## Materials and methods

### Cell lines and reagents

Human embryonic kidney 293 Jump In T-Rex cells (HEK293-JI) with doxycycline-inducible expression of human SLC39A5, SLC39A8, SLC39A10 and SLC39A14 were constructed by CeMM Research Center for Molecular Medicine of the Austrian Academy of Sciences, Austria (CeMM). Briefly, HEK293-JI cells were generated by transfection with a vector containing the human SLC gene with a TwinStrepTag and HA epitopes fused at C-terminal or N-terminal. Dulbecco’s modified Eagles medium (DMEM 1X + GlutaMAX + 4.5 g/L Glucose + Pyruvate), G418 were purchased from Sigma-Aldrich (Darmstadt, Germany). Hank’s Balanced Salt Solution (HBSS) and Blasticidin were obtained from ThermoFisher Scientific (Waltham, MA, USA). All other chemicals used in this study were purchased from commercial resources and were of analytical grade.

### Cell culture

HEK293-JI-ZIP-overexpressing cells were cultured in DMEM supplemented with 10% fetal calf serum (FCS), 100 µg/mL streptomycin and 100 IU/mL penicillin and split twice weekly to reach approximately 80% confluency. Prior to each experiment, cells were cultured for two passages in selection medium, i.e. culture medium supplemented with 2 mg/mL G418 and 5 µg/mL Blasticidin, to select for JumpIn-positive cells. When plated for functional assays, cells were grown in regular culture medium without the selection antibiotics.

### Immunoflourescence

HEK293-JI-ZIP-overexpressing cells were cultured in 24 well plates containing glass coverslips coated with Poly-L-Lysine Sigma Aldrich (Darmstadt, Germany) to confluency. Cells were then treated for 24 h with vehicle or doxycycline (1 µg/mL) to induce transgene overexpression. Cells were next fixed with 4% paraformaldehyde solution containing 0.3% Triton-X100. Samples were stained with primary anti-HA antibody from rat (Cat.#11867423001) from Merck (Darmstadt, Germany), followed by secondary anti-rat antibody coupled with Alexa Fluor 488 (Cat.# A-11006) from ThermoFisher Scientific (Waltham, MA, USA). Cells were counter-stained with Hoechst 33258 from Merck (Darmstadt, Germany).

### Ionomics

A detailed explanation and the rationale underlying the development of the ionomics protocol for sample preparation and ICP-MS analyses in metal uptake studies using HEK293 cells was described elsewhere (Dvorak et al. 2021). Briefly, HEK293-JI-ZIP-overexpressing cells were cultured in T175 cell culture flasks to confluency and treated 24 h with vehicle or doxycycline (1 µg/mL) to induce transgene overexpression. During the last 3 h prior to washing and lysis cells are incubated in a HBSS-based uptake buffer containing 12 mM HEPES, 0.8 mM Ascorbic acid, and 0.4% bovine-serum albumin at pH 7. Cells are incubated in uptake buffer added with 5 μM (NH_4_)_5_[Fe(C_6_H_4_O_7_)_2_] (ferric-ammonium citrate), 6 μM ZnCl_2_, 3 μM CuCl_2_, 6 μM MnCl_2_, 5 μM CoCl_2_ and 5 μM NiCl_2_ for metal uptake experiments. After, cells are thoroughly washed with an isotonic Tris/choline-chloride based wash buffer, to completely remove all extracellular ions and subsequently lysed with a Tris/choline-chloride/Triton X-100 based lysis buffer, not containing any of the measured ions. To the cell lysates diluted nitric acid and an internal standard mixture are added, the samples are analyzed by ICP-MS (Element 2, Thermo Fisher Scientific) using external calibration. An LC–MS method fit for purpose was applied. Concentrations outside the calibration range were extrapolated.

### Data analysis

All data were analyzed using Graphpad Prism 9.0 (Graphpad software Inc., San Diego, CA, USA). Data are presented as mean ± standard deviation of a least two biological replicates.

For ionomics analyses, element concentrations in the cell lysates were obtained with ICP-MS and normalized to total protein in the cytosolic fraction. Protein determination was performed using a BCA protein assay kit (ThermoFisher Scientific Cat. #23225). Statistical analyses were performed using Graphpad Prism 9.0. Normalized ion concentration values of DOX + vs DOX- samples were contrasted ina t-test and p-values (acquired from the t-test) were corrected for multiple testing using the Benjamini–Hochberg correction. The log2 fold change of each ion upon overexpression of a metal transporter was computed using the normalized ion concentrations. Differentially abundant ions were then detected by using a p-value threshold of < 0.01 and an absolute log2-fold change threshold of > 0.5.

### Phylogenetic analysis

We collected the sequences of ZIP transporters from seven representative species, including mouse (ie., *Mus musculus*); rat (ie., *Rattus norvegicus*); zebra fish (ie., *Danio rerio*); chicken (ie., *Gallus gallus*); fruit fly (ie., *Drosophila melanogaster*); C. elegans (ie., *Caenorhabditis elegans*); and humans (ie., *Homo sapiens)*. To identify the closest ZIP representative genes in all these organisms we used hierarchical orthoulogous groups as classified by the OMA database (Altenhoff et al. 2021) We also included the sequence of BbZIP, the *Bordetella bronchiseptica* homolog, whose structure has been solved (PDB; 5TSA). We aligned these sequences using BbZIP structure as a template through the software Promals3D (Pei et al. 2008), and filtered the resulting multiple sequence alignment using an in-house script. We used RaxML’s PROTGAMMAILG model, an amino acid evolutionary model incorporating a gamma rate heterogeneity with four categories, and estimated invariant sites (Stamatakis 2015). The resulting tree was explored using FigTree (v1.4.4).

### Supplementary Information

Below is the link to the electronic supplementary material.

Online Resource 1: Depicted are representative confocal microscopy images of HEK 293 JumpIn ZIP-5, -8, -10 and -14 overexpressing cells stained for HATag. ZIP expression can be seen in plasma membrane, nuclei are stained in blue. (JPG 352 kb)

Online Resource 2: The plots show the quantification of the concentrations measured in overexpressing cells (DOX) for the indicated elements analyzed by ICP-MS and expressed as log2 fold change against control (Ctrl) cells. Ion concentrations for each element were normalized to the total protein concentration in the respective cell lysate. The red dashed line represents the significance threshold, corresponding to a value of log2 fold change of 0.5 (at least 70% difference in +/- DOX) (N=3 biological replicates per group +/- DOX; **p<0.01). (JPG 516 kb)

Online Resource 3: The plots show the quantification of the concentrations measured in overexpressing cells (DOX) for the indicated elements analyzed by ICP-MS and expressed as log2 fold change against control (Ctrl) cells. Ion concentrations for each element were normalized to the total protein concentration in the respective cell lysate. The red dashed line represents the significance threshold, corresponding to a value of log2 fold change of 0.5 (at least 70% difference in +/- DOX) (N=3 biological replicates per group +/- DOX; **p<0.01; ***p<0.001). (JPG 778 kb)

## Data Availability

The data presented in this article may be provided by the corresponding author on reasonable request.
