# Correction: Functional characterization of SLC39 family members ZIP5 and ZIP10 in overexpressing HEK293 cells reveals selective copper transport activity

**DOI:** 10.1007/s10534-024-00615-z

**Published:** 2024-06-24

**Authors:** Marcello Polesel, Alvaro Ingles-Prieto, Eirini Christodoulaki, Evandro Ferrada, Cédric Doucerain, Patrick Altermatt, Michelle Knecht, Michael Kuhn, Anna-Lena Steck, Maria Wilhelm, Vania Manolova

**Affiliations:** 1 Vifor (International) AG, Wagistrasse 27a, 8952 Schlieren, Switzerland; 2 CeMM Research Center for Molecular Medicine of the Austrian Academy of Sciences, Lazarettgasse 14, 1090 Vienna, Austria

**Correction: BioMetals (2022) 36:227–237** 10.1007/s10534-022-00474-6

The article “Functional characterization of SLC39 family members ZIP5 and ZIP10 in overexpressing HEK293 cells reveals selective copper transport activity”, written by Marcello Polesel, Alvaro Ingles-Prieto, Eirini Christodoulaki, Evandro Ferrada, Cédric Doucerain, Patrick Altermatt, Michelle Knecht, Michael Kuhn, Anna-Lena Steck, Maria Wilhelm & Vania Manolova, was originally published Online First without Open Access. After publication in volume 36, issue 1, page 227–237 the authors decided to opt for Open Choice and to make the article an Open Access publication. Therefore, the copyright of the article has been changed to © the Author(s) 2024 and the article is forthwith distributed under the terms of the Creative Commons Attribution 4.0 International License, which permits use, sharing, adaptation, distribution and reproduction in any medium or format, as long as you give appropriate credit to the original author(s) and the source, provide a link to the Creative Commons licence, and indicate if changes were made.

The images or other third party material in this article are included in the article’s Creative Commons licence, unless indicated otherwise in a credit line to the material. If material is not included in the article’s Creative Commons licence and your intended use is not permitted by statutory regulation or exceeds the permitted use, you will need to obtain permission directly from the copyright holder.

To view a copy of this licence, visit http://creativecommons.org/licenses/by/4.0/.

