# Supplementary figures and images for: Functional characterization of SLC39 family members ZIP5 and ZIP10 in overexpressing HEK293 cells reveals selective copper transport activity
Source: Biometals. 2022 Dec 1;36(1):227–37. doi: 10.1007/s10534-022-00474-6 (PMC11196296; doi:10.1007/s10534-022-00474-6)

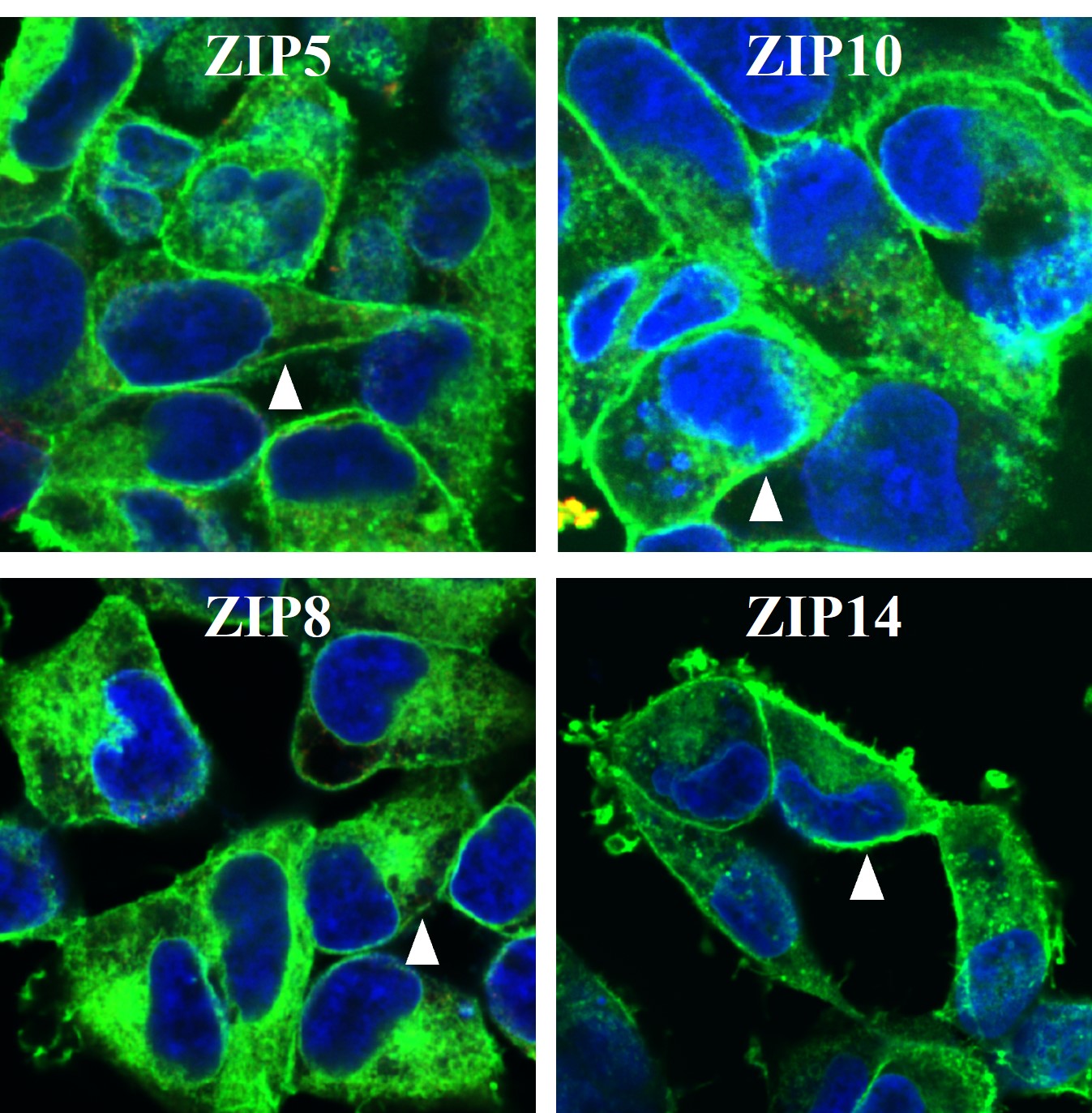

Supplement: Supplementary file 1 — Online Resource 1: Depicted are representative confocal microscopy images of HEK 293 JumpIn ZIP-5, -8, -10 and -14 overexpressing cells stained for HATag. ZIP expression can be seen in plasma membrane, nuclei are stained in blue. (JPG 352 kb) [file 10534_2022_474_MOESM1_ESM.jpg]

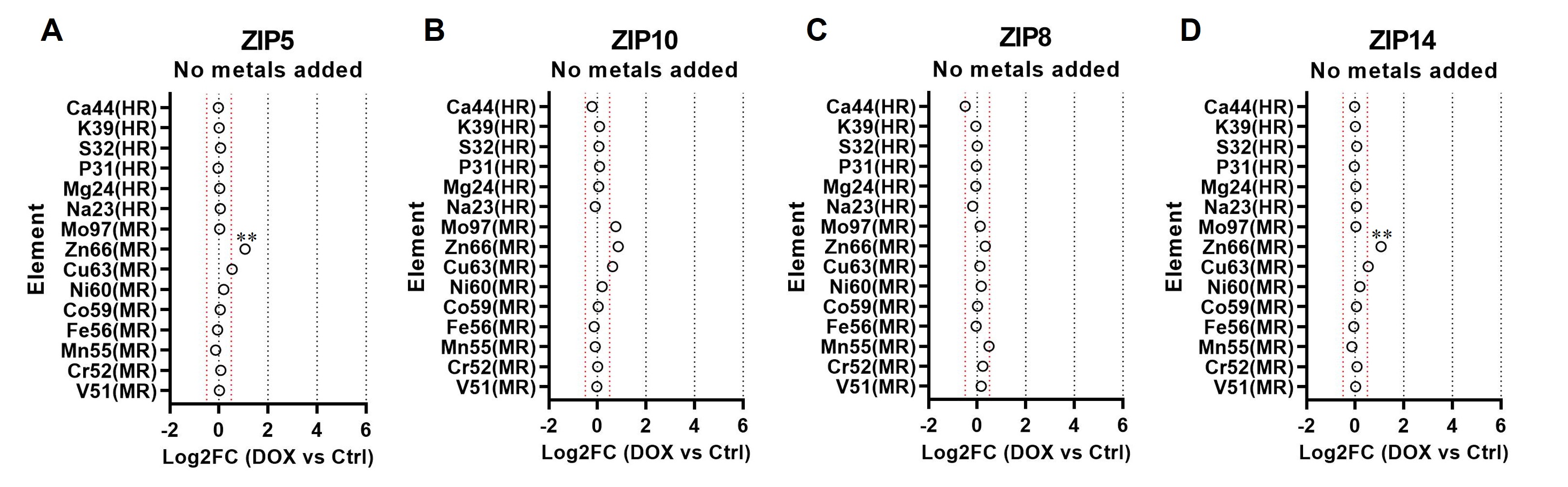

Supplement: Supplementary file 2 — Online Resource 2: The plots show the quantification of the concentrations measured in overexpressing cells (DOX) for the indicated elements analyzed by ICP-MS and expressed as log2 fold change against control (Ctrl) cells. Ion concentrations for each element were normalized to the total protein concentration in the respective cell lysate. The red dashed line represents the significance threshold, corresponding to a value of log2 fold change of 0.5 (at least 70% difference in +/- DOX) (N=3 biological replicates per group +/- DOX; **p<0.01). (JPG 516 kb) [file 10534_2022_474_MOESM2_ESM.jpg]

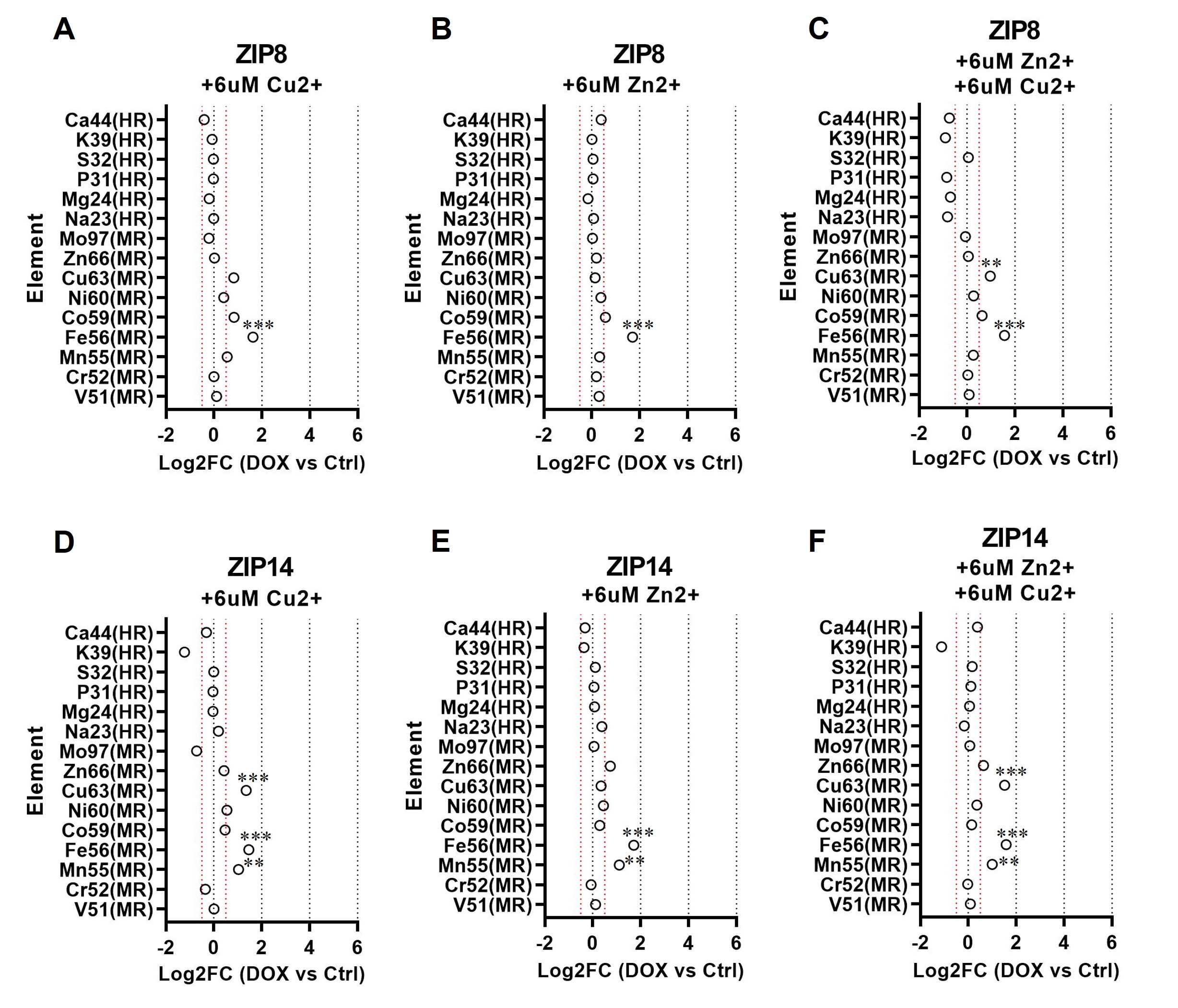

Supplement: Supplementary file 3 — Online Resource 3: The plots show the quantification of the concentrations measured in overexpressing cells (DOX) for the indicated elements analyzed by ICP-MS and expressed as log2 fold change against control (Ctrl) cells. Ion concentrations for each element were normalized to the total protein concentration in the respective cell lysate. The red dashed line represents the significance threshold, corresponding to a value of log2 fold change of 0.5 (at least 70% difference in +/- DOX) (N=3 biological replicates per group +/- DOX; **p<0.01; ***p<0.001). (JPG 778 kb) [file 10534_2022_474_MOESM3_ESM.jpg]
